# Generation of a monoclonal antibody against the glycosylphosphatidylinositol-linked protein Rae-1 using genetically engineered tumor cells

**DOI:** 10.1186/1480-9222-16-3

**Published:** 2014-02-04

**Authors:** Jiemiao Hu, Long T Vien, Xueqing Xia, Laura Bover, Shulin Li

**Affiliations:** 1 Department of Pediatrics–Research, Unit 0853, The University of Texas MD Anderson Cancer Center, 1515 Holcombe Blvd., Houston, TX 77030, USA; 2 Department of Immunology, The University of Texas MD Anderson Cancer Center, Houston, TX, USA

**Keywords:** GPI-anchored protein Rae-1, Monoclonal antibody, Hybridomas, Streamlined screening strategy

## Abstract

**Background:**

Although genetically engineered cells have been used to generate monoclonal antibodies (mAbs) against numerous proteins, no study has used them to generate mAbs against glycosylphosphatidylinositol (GPI)-anchored proteins. The GPI-linked protein Rae-1, an NKG2D ligand member, is responsible for interacting with immune surveillance cells. However, very few high-quality mAbs against Rae-1 are available for use in multiple analyses, including Western blotting, immunohistochemistry, and flow cytometry. The lack of high-quality mAbs limits the in-depth analysis of Rae-1 fate, such as shedding and internalization, in murine models. Moreover, currently available screening approaches for identifying high-quality mAbs are excessively time-consuming and costly.

**Results:**

We used Rae-1–overexpressing CT26 tumor cells to generate 60 hybridomas that secreted mAbs against Rae-1. We also developed a streamlined screening strategy for selecting the best anti–Rae-1 mAb for use in flow cytometry assay, enzyme-linked immunosorbent assay, Western blotting, and immunostaining.

**Conclusions:**

Our cell line–based immunization approach can yield mAbs against GPI-anchored proteins, and our streamlined screening strategy can be used to select the ideal hybridoma for producing such mAbs.

## Introduction

Since 1975, when Kohler and Milstein first reported producing monoclonal antibodies (mAbs) by generating and isolating hybridoma cells [1], mAbs have been broadly used in research and therapy. At the same time, mAb production methods have been improving.

Rae-1α, -1β, -1γ, -1δ, and -1ϵ are mouse NKG2D ligands that have been reported to have very low expression levels in the normal tissues of adult mice [2]. However, the expression of these ligands can be upregulated in infectious tissues or tumor cell lines [3]. All Rae-1 family members have GPI anchors that lack cytoplasmic domains. To produce an mAb targeting the mouse NKG2D ligand Rae-1, one must overcome the insolubility problem and the post-translational modifications of the Rae-1 protein.

In traditional mAb production, the first step is to produce the antigen of interest. For decades, live cells whose surfaces endogenously display certain proteins have been used as antigens to induce immune responses in animals. In the 1980s, as eukaryotic vectors were being developed, cells stably transfected with non-endogenous proteins were used as antigens to produce antibodies [4]. Some groups have reported that live mammalian cells that express certain proteins on their surfaces could be used to immunize animals to generate mAbs [4,5]. In the present study, our objective was to use a mouse cell line stably transfected with *pBMN Rae -1 GFP* to show that cell-based immunization can yield hybridomas to produce mAbs against the glycosylphosphatidylinositol (GPI)-linked protein Rae-1.

In the present study, we applied a novel strategy of antigen preparation and animal immunization to develop an anti–Rae-1 mAb. We stably transfected full-length Rae-1δ into murine CT26 cells using a retrovirus system, the vector transfected cells as control, and then immunized animals with the antigen-expressing cells or the control vector transfected cells. Thus, we describe how to use stably transfected cells as the GPI antigen to immunize animals to generate mAbs that could be used for enzyme-linked immunosorbent assay (ELISA), Western blotting, flow cytometry, immunofluorescence staining, immunohistochemistry, and potentially therapeutic purposes.

## Materials and methods

### Cell culture and establishment of a cell line stably transfected with Rae-1

The cancer cell lines CT26, TC1, B16F10, LLC, K7M3, and YAC-1 were obtained from American Type Culture Collection (Rockville, MD, USA). CT26, TC1, K7M3, B16F10, and LLC cells were grown in Dulbecco's modified Eagle’s medium (Mediatech, Inc., Manassas, VA, USA) supplemented with glutamine, heat-inactivated 10% fetal calf serum, and 10 U/ml penicillin and streptomycin. YAC-1 cells were grown in RPMI-1640 medium (Mediatech, Inc.) supplemented with heat-inactivated 10% fetal calf serum and 10 U/ml penicillin and streptomycin.

The murine gene Rae-1δ (Open Biosystems) was subcloned into a pBMN–green fluorescent protein (GFP) plasmid. Retroviruses were produced by transfecting mRae-1δ/pBMN-GFP constructs into Phoenix-ECO packaging cells. CT26 cells were infected with the retrovirus-containing supernatant derived from the transduced HEK293 cells. Cell colonies were expanded from a single cell expressing GFP. Both Rae-1δ/GFP and GFP-positive CT26 cells were confirmed using flow cytometry.

### Mouse immunization

Stable transfected cells were washed twice in phosphate-buffered saline (PBS), counted, suspended in 100 μl of sterile PBS, and then transferred to a 0.5-ml tuberculin syringe. Six- to seven-week-old BALB/C mice were injected with 35 × 10^6^ cells in a 50-μl volume in each foot. The mice received injections every 3 days for 18 days (6 injections total). On day 18, the mice were humanely killed, and B cells were isolated from lymph nodes for fusion.

### Myeloma cells expansion

One week before fusion was to be performed, we began growing SP2/0-Ag14 myeloma cells in a 10-cm petri dish containing RPMI medium supplemented with 10% FBS to ensure that 1 × 10^8^ cells would be available for fusion.

### Mouse lymph nodes harvest

For the mouse lymph node harvest, we first prepared RPMI medium containing 10% FBS, 1× PN/SM and 1× hypoxanthine, aminopterin, and thymidine (HAT) medium, and we prewarmed 50% polyethylene glycol (PEG; Sigma) in a 37°C incubator. We then euthanized the mice and aseptically harvested the lymph nodes. We transferred the lymph nodes into a sterile 10-cm petri dish containing 10 ml of serum-free RPMI medium. We used forceps to manipulate the lymph nodes to release cells and transferred the lymphocyte suspension to a sterile 50-ml conical centrifuge tube that we then filled with serum-free RPMI medium. We washed the cells 2 times with serum-free RPMI medium. To harvest the Sp2/0-Ag14 myeloma cells, we transferred the cells into 50-ml conical centrifuge tubes and centrifuged them at 1150 rpm for 3 min at room temperature. After aspirating and discarding the supernatant, we resuspended the SP2/0-Ag14 cells in serum-free RPMI medium and washed them 2 times. We used a hemacytometer and staining with trypan blue to count the cells in each suspension and assess their viability.

### Cell fusion for mAbs

On the day fusion was performed, mouse lymph nodes were harvested to obtain the lymphocytic cells. Lymphocytes and myeloma cells were harvested, washed, and then mixed together. Cell fusion was performed in the presence of polyethylene glycol (PEG). The resulting pellet was harvested and placed in tissue culture plates. After incubation with hypoxanthine, aminopterin, and thymidine (HAT) medium and feeding for 10 days, the hybridomas were ready for screening.

Lymphocytes and Sp2/0-Ag14 myeloma cells were mixed in a 50-ml conical tube at a ratio of 1:0.8. The tube was then filled with serum-free RPMI medium, and the cell mixture was subjected to centrifugation at 1350 rpm for 5 min at room temperature. After the supernatant was aspirate and discarded, 1 ml of sterile PEG was added to the cell pellet. The cell pellet was then agitated for 45 sec, and 40 ml of prewarmed serum-free RPMI medium was added to stop the reaction. The mixture was then subjected to centrifugation at 1150 rpm for 5 min at room temperature. The supernatant was aspirated, and the cell pellet was resuspended in HAT medium. The cells were then placed in 96-well flat-bottom plates.

### ELISA

Costar EIA/RIA plates (Fisher Scientific, Hampton, NH) were coated with 20 ×10^6^ cells/plate and allowed to dry overnight before storage in a -20°C freezer until use. For ELISA, the cells were washed with PBS containing 0.05% Tween 20 (PBST) 3 times and blocked by incubation in PBST containing 2% bovine serum albumin for 1 hour at room temperature. Culture supernatant (100 μl) was then added, and the cells were incubated for 1 hour at room temperature and then washed with PBST 3 times. Goat anti-mouse immunoglobulin G (IgG) Fc, horseradish peroxidase (HRP) conjugate (100 μl; Jackson Immunoresearch: 115-035-071) was then added, and the cells were incubated at room temperature for 1 hour and washed 5 times with PBST before the substrate was added. Absorbance was read at 450 and 620 nm.

### Western blotting

Different amounts of Rae-1β recombinant protein were loaded onto 10% sodium dodecyl sulfate–polyacrylamide gel and transferred to nitrocellulose membranes using the iBlot gel transfer device (Invitrogen, Grand Island, NY). The membranes were blotted with anit–Rae-1 primary antibody and HRP-conjugated goat anti-mouse secondary antibody (Santa Cruz Biotechnology, Dallas, TX) to detect the protein of interest.

### Flow cytometry

Cells were gently trypsinized and washed once with serum-containing media. The cell pellets were washed with cold PBS free of Ca^2+^ and Mg^2+^ and then resuspended in 100 μl of PBS. The cells were stained with the indicated primary and secondary antibodies for 30 min at 4°C. The expression of the indicated genes was analyzed using a FACSCalibur flow cytometer (BD Biosciences).

### DNA transfection

CT26 cells (1 × 10^6^) were transfected with 1 μg of plasmid DNA using the X-tremeGENE HP DNA transfection reagent (Roche Diagnostics, Indianapolis, IN).

### Immunofluorescence staining

Cells were seeded on coverslips in 12-well plates (1×10^5^ cells/well). The next day, the cells were washed in 1× HEPES-buffered Hank’s balanced salt solution (HEPES/HANKS) buffer and then fixed in 1% paraformaldehyde for 30 min. After the paraformaldehyde was removed carefully and discarded, the cells were rinsed in PBS for 5 min 3 times. The cells were blocked in 1% goat serum in PBS for 1 h, the primary antibody was added, and the cells were incubated overnight at 4°C or for 2 h at room temperature if needed. After the cells were washed with PBS for 5 min 3 times, the secondary antibody was added, and the cells were incubated for 60 min room temperature. The cells were then washed with PBS for 5 min 3 times, rinsed with water, mounted on slides, and coated with anti-fade reagent (Life Technologies, Carlsbad, CA). The slides were stored in the dark before they were observed under a fluorescence microscope.

### Tumor inoculation and frozen tissue section preparation

Cells stably transfected with Rae-1 (CT26–Rae-1 cells) and control cells (CT26-GFP cells) were subcutaneously injected into BALB/c mice (2 × 10^5^ cells/mouse). Fourteen days after injection, the mice were humanely killed and their tumors harvested. The tumors were frozen in (optimum cutting temperature) OTC solution, and tissues sections were cut and mounted on glass slides.

### Immunohistochemistry staining

Frozen tumor sections were sequentially fixed with cold acetone, acetone plus chloroform (1:1), and acetone. Tissue sections were blocked with blocking buffer (5% normal horse serum and 1% normal goat serum in PBS) and then incubated with the primary antibody overnight at 4°C. The next day, the tissues were incubated with the secondary antibody for 1 hour at room temperature. Nuclei were counterstained with hematoxylin (Sigma-Aldrich, St. Louis, MO).

## Results

### Establishment of CT26–Rae-1 cells

**Figure 1:**
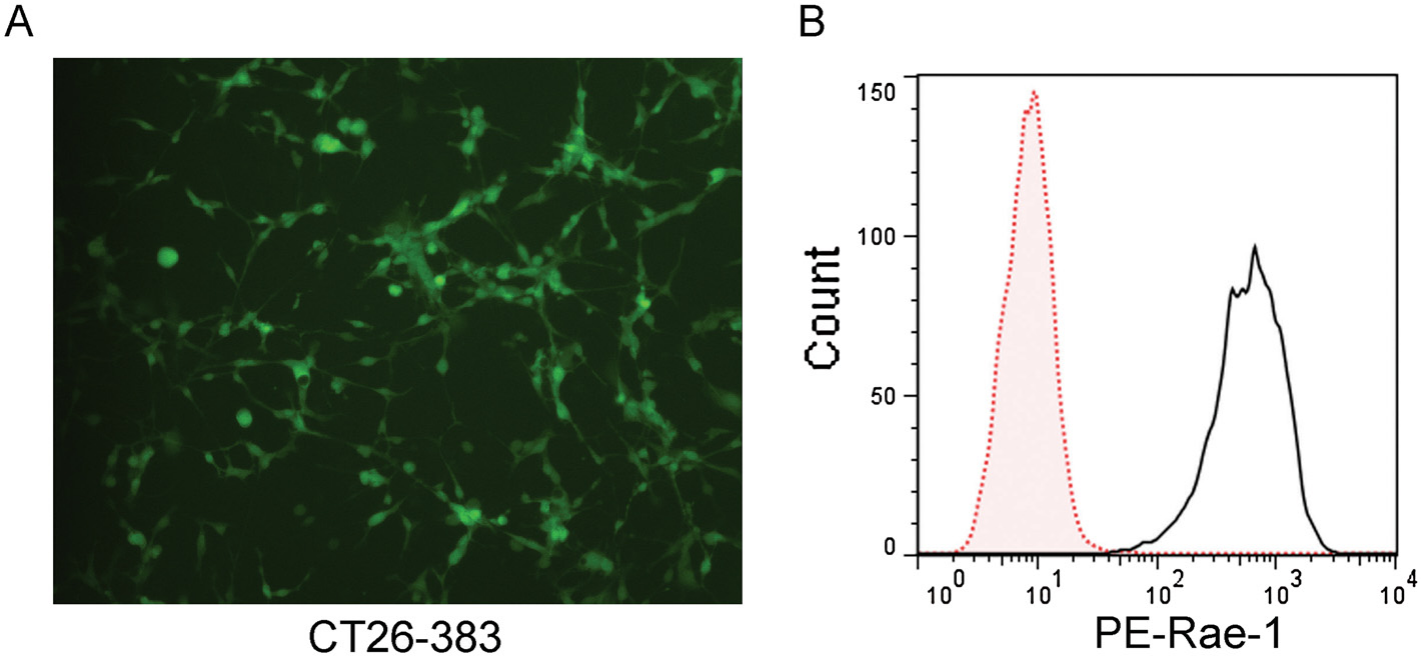
**Confirmation of stable transfection of Rae-1 into CT26-383 cells. A**. Fluorescence microscopy image of Rae-1ϵ/GFP engineered CT26-383 tumor cells. **B**. CT26-383 cells’ expression of Rae-1 was assessed using flow cytometry. Red dot: CT26-GFP control cells. Black solid: CT26-383 cells.

Murine colon cancer cells (CT26 cells) were stably transfected with the full-length Rae-1δ gene via the retrovirus vector pBMN-GFP. Control cells were CT26 cells transfected with the pBMN-GFP vector alone. GFP-positive colonies were selected and expanded. Rae-1 transfection was confirmed by observation under a fluorescence microscope. More than 90% of the cells were GFP-positive (Figure 1A). Flow cytometry revealed that, compared with the control cells, the Rae-1–transfected CT26 cells had a dramatically higher level of Rae-1 expression on the cell surface (Figure 1B). Therefore, Rae-1–positive CT26 cells were ready for animal inoculation.

### mAbs screening

**Figure 2:**
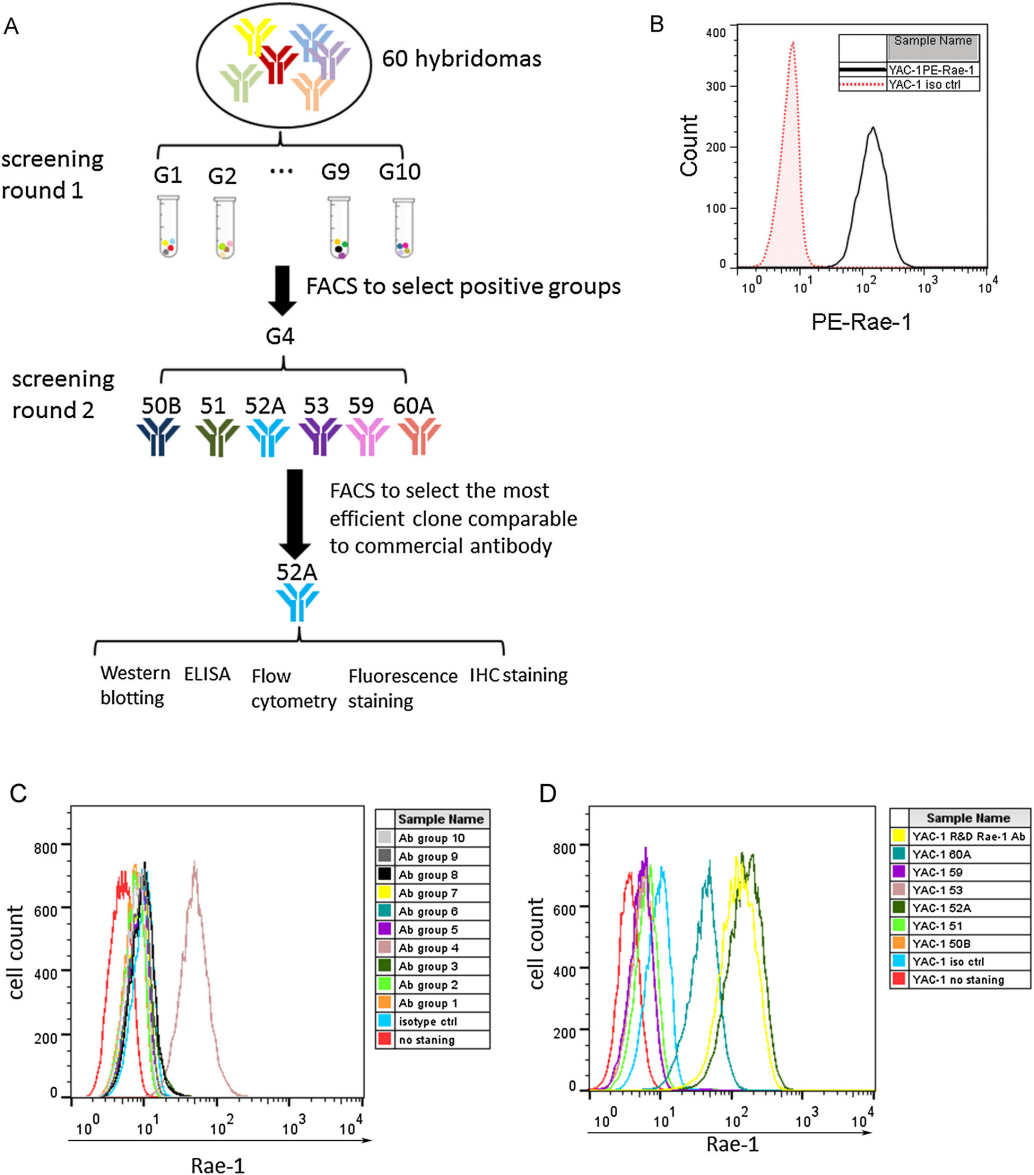
**Screening of the most sensitive mAbs for Rae-1 detection. A**. Flow chart of the streamlined screening process. **B**. Rae-1 expression in YAC-1 cells was confirmed using a commercial phycoerythrin (PE)-anti–Rae-1 antibody. Red dot: isotype control. Black solid: PE-anti–Rae-1 antibody. **C**. The streamlined approach and FACS analysis were used to screen 60 subclones of hybridomas producing anti–Rae-1 mAbs. YAC-1 cells were not stained, stained with isotype control antibody, or stained with culture containing a mixture of different groups’ anti–Rae-1 mAb–producing hybridoma cells. Flow cytometry was performed to determine whether the anti–Rae-1 mixture recognizes Rae-1 expression on YAC-1 cells. **D**. YAC-1 cells were not stained, stained with isotype control antibody, stained with PE-anti–Rae-1 antibody, or stained with each anti–Rae-1 mAb–conditioned medium of group 4. Flow cytometry was performed to assess Rae-1 expression levels.

Sixty different anti–Rae-1 subclones that could recognize Rae-1–expressing cells but not the control vector–transfected cells were selected for screening. For the first round of screening for the effective antibodies, 10 groups of 6 antibodies each were subjected to a single flow cytometry assay. We used fluorescence-activated cell sorting (FACS) analysis of a different Rae-1–positive cell line, YAC-1, to select the best group of hybridomas among the 10 groups of anti–Rae-1 hybridoma culture medium mixture. Because this method quickly identifies the best group of hybridomas, it was selected as the first step of the streamline analysis (Figure 2A). A commercial anti–Rae-1 antibody was used as the control (Figure 2B). We found that antibodies from the hybridoma culture medium mixture in group 4 could effectively stain Rae-1 on the YAC-1 cell surface, whereas antibodies in other groups either detected a low level of Rae-1 expression or failed to detect Rae-1 expression in the same YAC-1 cells (Figure 2C). This single step eliminated 90% of hybridoma cell lines from further consideration.

The second round of screening, we used FACS to compare the detection sensitivity of the individual mAbs in group 4 (Figure 2A). Compared with a commercial anti–Rae-1 antibody, two subclones in group 4, 52A and 60A, could detect Rae-1 expression on YAC-1 cells with high sensitivity (Figure 2D). Our results suggested that the 52A antibody is as efficient as the commercial antibody and that the 60A antibody can be used for flow cytometry. This step excluded the other 8 hybridomas from further consideration.

### Screening confirmed by ELISA

**Figure 3:**
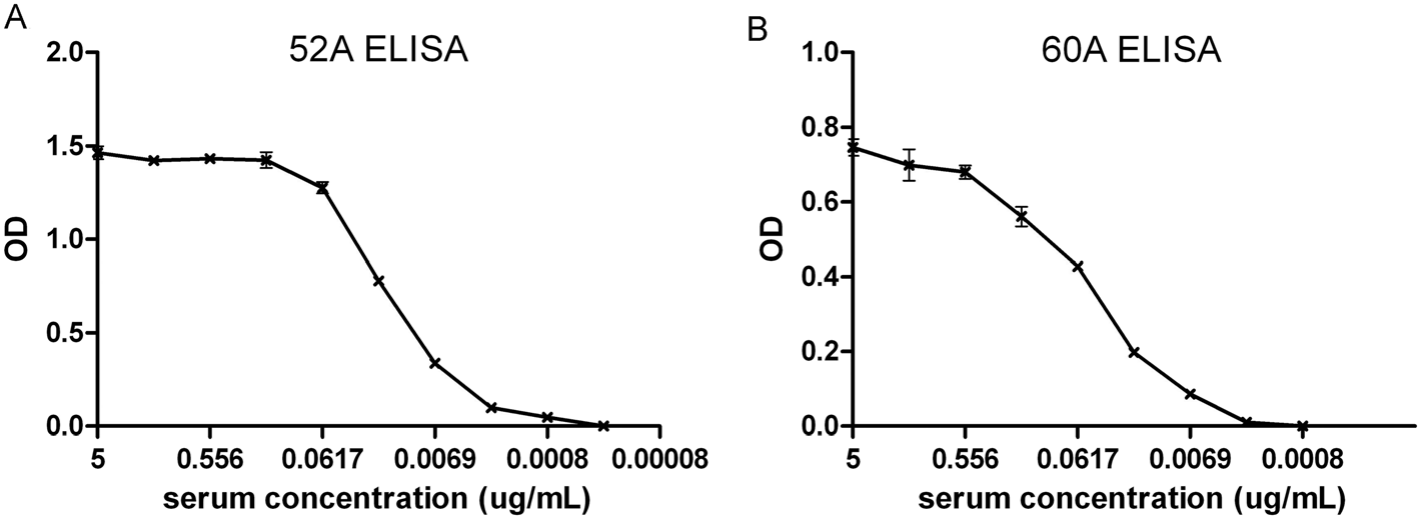
**The anti-Rae-1 mAb subclones 52A and 60A recognize Rae-1 expression in ELISA.** The anti–Rae-1 mAb subclones 52A **(A)** and 60A **(B)** were diluted, and ELISA was performed to detect Rae-1–expressing cells.

ELISA was used to test the ability of the purified anti–Rae-1 52A and 60A subclone serums to recognize Rae-1–expressing cells. The 52A and 60A serums were diluted to different concentrations; even at very low concentrations, the serums had strong reaction with Rae-1–expressing cells (Figure 3A, 3B). We concluded that the anti–Rae-1 mAb subclones 52A and 60A—especially 52A—had supreme binding efficiency with Rae-1–expressing cells.

### Application of the 52A anti–Rae-1 mAb

**Figure 4:**
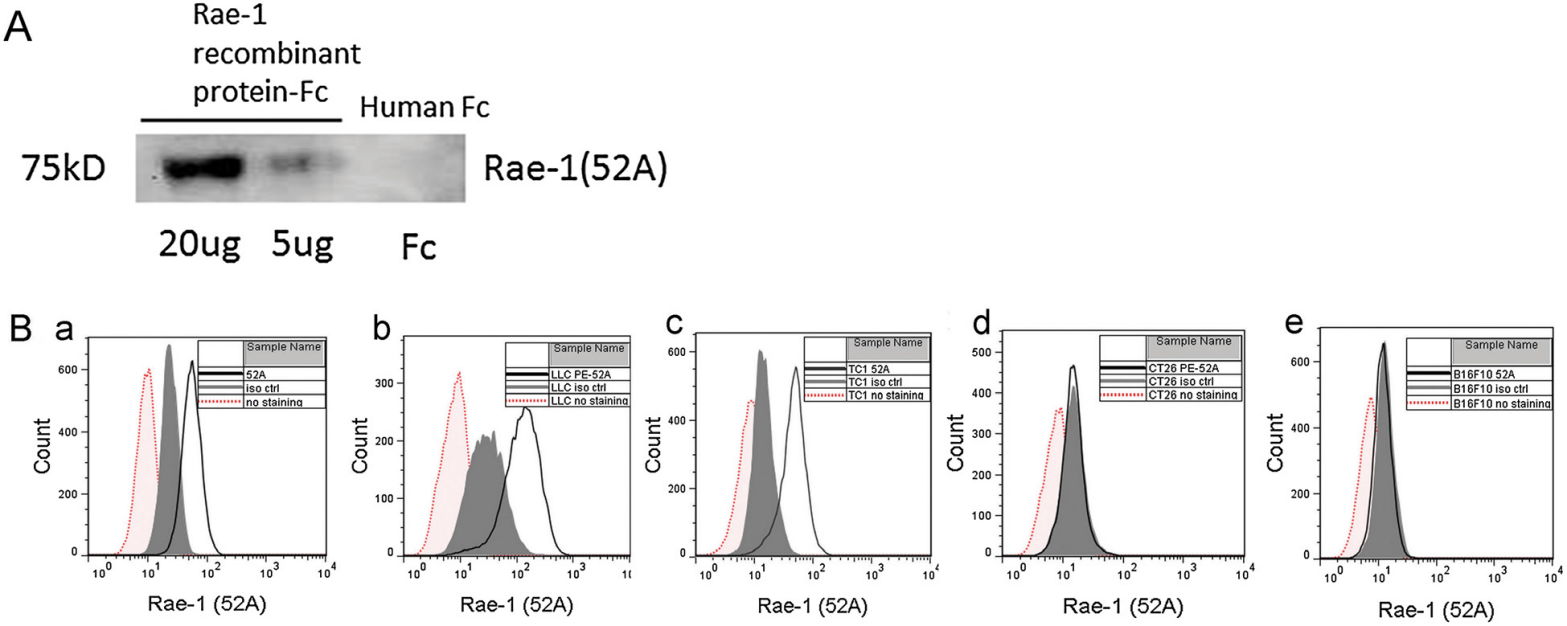
**Application of the anti–Rae-1 mAb in Western blotting and flow cytometry. A**. Detection of Rae-1 recombinant protein in Western blotting using the 52A mAb. Rae-1b recombinant protein (20 μg and 5 μg) and control human IgG-Fc (20 μg) were loaded onto 10% sodium dodecyl sulfate–polyacrylamide gel. In the Western blot assay, the primary antibody was the 52A anti–Rae-1 mAb, and the secondary antibody was HRP goat anti-mouse IgG. **B**. Detection of Rae-1 expression levels in multiple murine cancer cell lines using the 52A anti–Rae-1 mAb. Murine tumor cells were not stained, stained with isotype control, or stained with the 52A anti–Rae-1 mAb. Rae-1 expression levels were assessed using flow cytometry. **a**, K7M3 cells; **b**, LLC cells; **c**, TC1 cells; **d**, CT26 cells; **e**, B16F10 cells.

To determine the efficiency of the 52A anti–Rae-1 antibody, we loaded Rae-1β–Fc recombinant protein (20 μg and 5 μg) onto 10% sodium dodecyl sulfate–polyacrylamide gel for immunoblotting detection. We also loaded human IgG-Fc (20 μg) was as a negative control. Using the 52A anti–Rae-1 antibody as a primary antibody and a HRP-conjugated goat anti-mouse IgG secondary antibody, we found that Rae-1β recombinant protein, but not the negative control, could be specifically detected in a dose-dependent manner (Figure 4A).

Multiple cancer cell lines express Rae-1. To determine the level of Rae-1 expression on the murine cancer cell lines CT26, B16F10, LLC, TC1, and K7M3, we incubated cells with the 52A anti–Rae-1 mAb and then fluorescein isothiocyanate (FITC)-conjugated goat anti-mouse IgG and performed flow cytometry. We found that the CT26 and B16F10 cell lines were Rae-1–negative cell lines and the LLC, TC1, and K7M3 cell lines were Rae-1–positive (Figure 4B).

**Figure 5:**
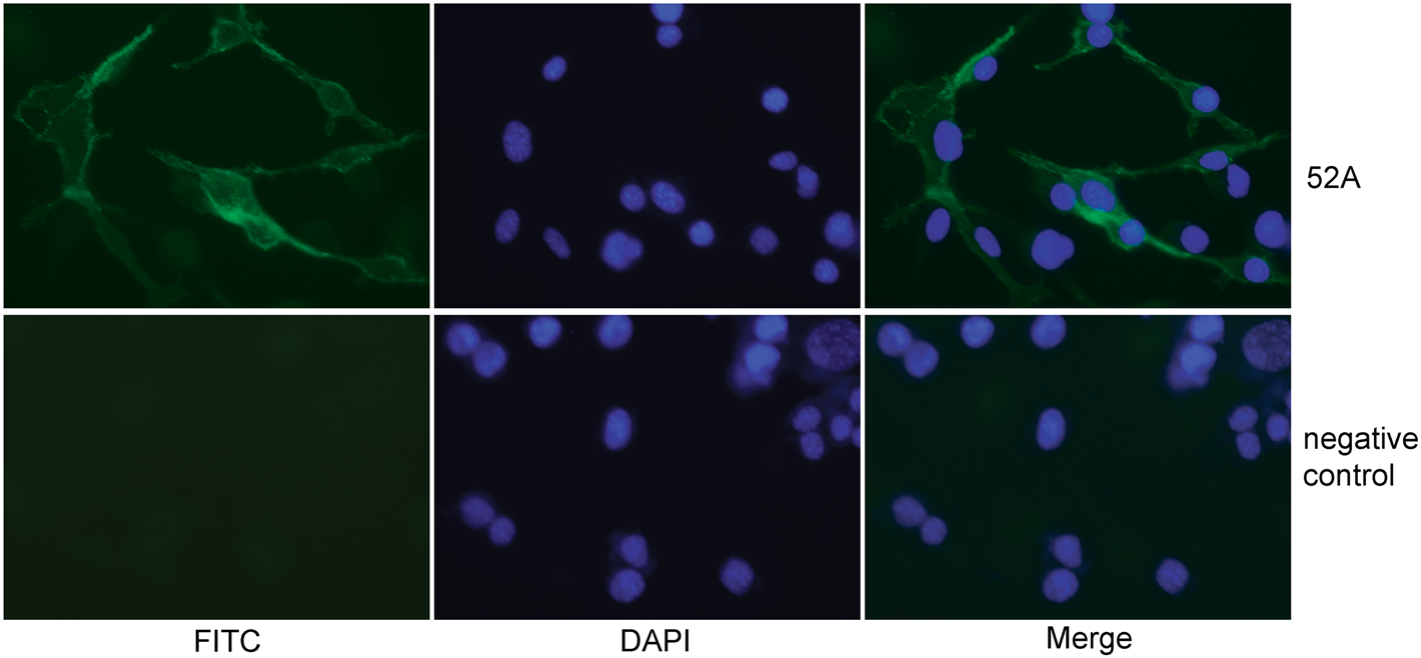
**Application of the anti–Rae-1 mAb in cell immunofluorescence staining.** Rae-1–expressing CT26 cells were stained with the 52A anti–Rae-1 mAb and then stained with FITC-conjugated goat anti-mouse IgG secondary antibody or secondary antibody alone as a negative control. FITC and DAPI signals were visualized using fluorescence microscopy.

The Rae-1 mAb could also be used for immunofluorescence staining. We cultured Rae-1–overexpressing cells on coverslips in a 12-well plate, stained the cells with the 52A anti–Rae-1 mAb and then with GFP-conjugated anti-mouse secondary antibody or secondary antibody alone (negative control). The coverslips were mounted onto glass slides with 4',6-diamidino-2-phenylindole (DAPI) containing anti-fade mounting buffer. Compared with the negative controls, the cells stained with the 52A mAb showed positive staining for Rae-1 (Figure 5). This suggests that the 52A anti–Rae-1 mAb recognizes Rae-1–expressing cells and thus could be used to detect Rae-1 localization in tumor cells.

**Figure 6:**
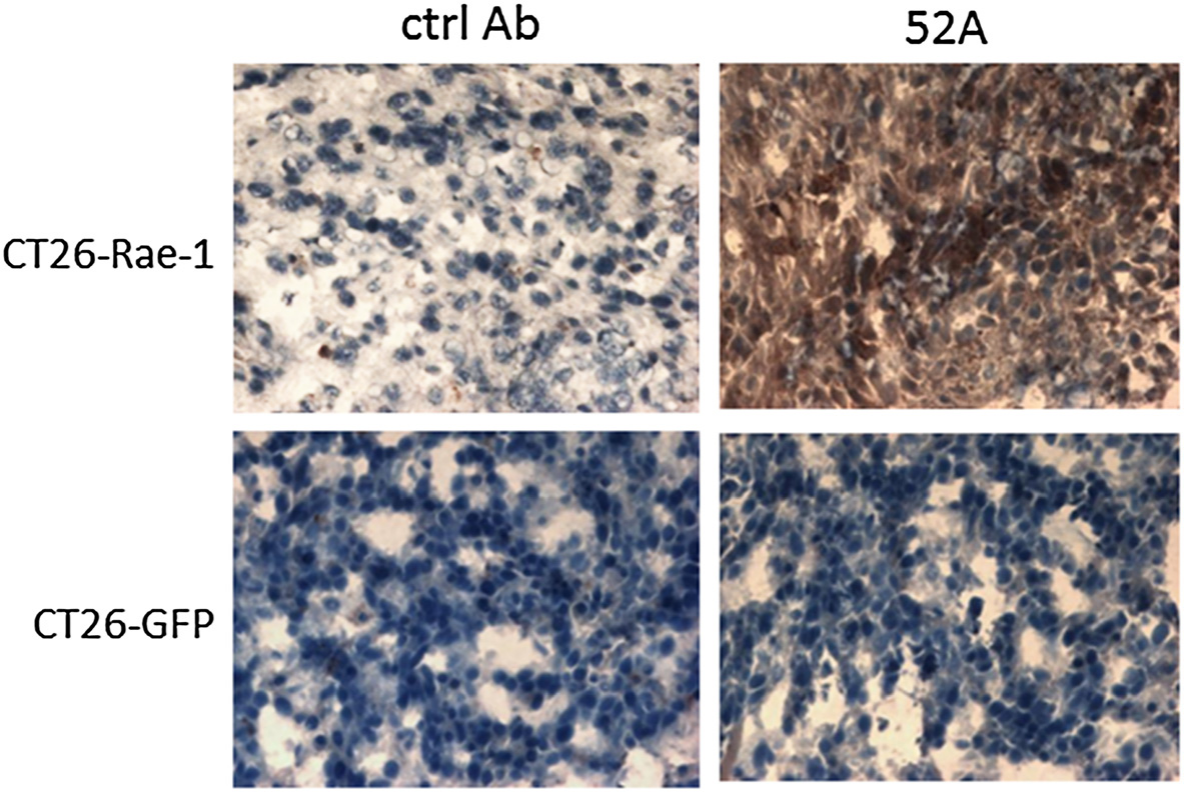
**Application of the anti–Rae-1 mAb in immunohistochemistry staining of tissue sections.** CT26–Rae-1 and CT26-GFP frozen tumor sections were stained with the 52A anti–Rae-1 mAb and then stained with HRP-conjugated goat anti-mouse secondary antibody or secondary antibody alone.

We also sought to determine whether the 52A anti–Rae-1 antibody could be used in immunohistochemistry staining of frozen tissue sections, which would facilitate identifying Rae-1’s function *in vivo*. The anti–Rae-1 antibody showed positive staining on CT26–Rae-1 tumor sections but not control CT26-GFP tumor sections (Figure 6). Our results demonstrated that the 52A anti–Rae-1 mAb could be applied in immunohistochemistry staining.

## Discussion

In this study, we used live cells that stably overexpressed the transfected mouse NKG2D ligand Rae-1as the antigen to generate an mAb that can be used effectively in Western blotting, flow cytometry, ELISA, and immunofluorescence and immunohistochemistry staining (Figures 3, 4, 5 and 6).

We also generated a streamlined screening process to exclude hybridomas that produce less-sensitive mAbs and select the hybridoma that produces a versatile mAb for multiple applications (Figure 2A). The mAb we selected, 52A anti-Rae-1, can bind Rae-1ϵ, which was used to engineer the CT26 cell line, as well as Rae-1β, whose recombinant protein was used for Western blot analysis. Most likely, this antibody recognizes all Rae-1 subtypes owing to the subtle difference. However, this streamlined selection process does not consider antibodies in the condition medium, which may lead to some mAbs that have a high detection power being overlooked because of their low concentrations. To avoid this concern, investigators could seed equal numbers of hybridomas, which in theory would yield similar concentrations of the anti–Rae-1 mAb. Of course, some hybridomas may have low rates of mAb secretion which would lower the total concentration of mAb in the condition medium. Most investigators likely will not need to address this concern, however, because they would likely want to exclude hybridomas that have low secretion efficiency during the initial screening step. Another caveat of this streamlined screening is that, because FACS is used in the initial screening step, the best antibody for Western blotting or immunostaining may be missed. Depending on the primary purpose of the study in which the mAb will be used, different applications could be used for the initial screening step to address this concern.

Various antigens can be used to generate mAbs, including recombinant proteins, peptides, and others [6,7]. The system most commonly used to induce protein expression is the prokaryotic expression system, which usually utilizes *E. coli*. A tag such as Glutathione S-transferase (GST) or polyhistidine (His) is introduced to facilitate purification. The advantages of this system are its high efficiency and low cost. However, a bacteria-based system is less likely to produce folded and active proteins, especially transmembrane proteins, which tend to have highly species-specific post-translational modifications that could be crucial for mAb generation. It is also difficult to use bacteria-based systems to produce a sufficient amount (~10 mg) of the soluble forms of proteins embedded in the cell membrane and proteins larger than 60 kD [8].

Given the limitations of bacteria-based systems, yeast, insect [9] and mammalian expression systems [10,11] are popular. First, eukaryotic systems have the advantage of conforming to the complexity of proteins that are correctly assembled and biologically functional. Second, native eukaryotic proteins tend to form insoluble inclusion body aggregates in bacteria. Also, post-translational modifications that are highly related to protein function, such as glycosylation, phosphorylation, and farnesylation, are added to proteins in eukaryotic system but not to proteins in bacteria systems [12]. However, using eukaryotic system to induce protein expression for mAb production is very time-consuming and costly and yields only a small amount of purified proteins, which makes producing at least 10 mg of purified proteins for immunization quite challenging. Another issue with eukaryotic systems that we have experienced is that because tags are introduced to plasmid DNA to facilitate protein purification, antibodies targeting the tags but not the proteins of interest are likely to be generated.

Peptides are also widely used as the epitope for mAb generation. Phage display and a few established databases are used to design peptide epitopes, but this approach invariably fails to produce antibodies that target folded proteins.

Introducing the protein of interest into eukaryotic cells guarantees the overexpression of correctly folded and functional antigen protein, which is very difficult to achieve using a bacteria-based system, especially for glycoprotein. In immunizing animals, using a protein that displays the natural conformation as the antigen is more than likely to generate an effective mAb. Because antigen proteins are expressed on the cell surface, the protein solubility issue is avoided altogether.

The successful generation of this mAb will benefit our current study of Rae-1 regulation. Other potential uses of the antibody may include targeted therapy, cell therapy, and imaging study applications.

## Competing interests

The authors declare that they have no competing interests.

## Authors’ contributions

JH carried out the screening of Rae-1 specific hybridoma subclones, performed WB, Flow cytometry, Immunofluorescence and Immunohistochemistry staining, also drafted this manuscript; LV generated the hybridomas, selected the potential hybridoma subclones targeting to Rae-1, performed ELISA to confirm the efficiency, and harvested ascites from mice; XX developed pBMN-Rae-1 construct, established CT26-Rae-1 and CT26-GFP stable transfected cell lines; LB involved in the design and part of writing in Material and Methods; SL contributed to the design, interpretation of data, editing and finalization of the manuscript. All authors read and approved the final manuscript.
